# Comparison of [^99m^Tc]Tc-tilmanocept with [^99m^Tc]Tc-sulphur colloids and [^99m^Tc]Tc-albumin colloids for sentinel lymph node detection in patients with cutaneous malignancies of the head

**DOI:** 10.1007/s00259-022-06017-y

**Published:** 2022-10-28

**Authors:** Mark Ooms, Dirk von Mallek, Hans-Jürgen Kaiser, Frank Hölzle, Felix M. Mottaghy, Ali Modabber

**Affiliations:** 1 Department of Oral and Maxillofacial Surgery, University Hospital RWTH Aachen, Pauwelsstraße 30, 52074 Aachen, Germany; 2 Department of Nuclear Medicine, University Hospital RWTH Aachen, Pauwelsstraße 30, 52074 Aachen, Germany; 3 Department of Radiology and Nuclear Medicine, Maastricht University Medical Center (MUMC+), P. Debyelaan 25, 6229 HX Maastricht, The Netherlands

**Keywords:** Sentinel lymph node biopsy, Lymphoscintigraphy, SPECT/CT, [^99m^Tc]Tc-tilmanocept, [^99m^Tc]Tc-sulphur colloid, [^99m^Tc]Tc-albumin colloid

## Abstract

**Purpose:**

Sentinel lymph node (SLN) biopsy is a staging procedure in the management of cutaneous malignancies of the head. The ideal radiopharmaceutical is controversial. This study aimed to compare [^99m^Tc]Tc-tilmanocept (TcTM) with [^99m^Tc]Tc-sulphur colloid (TcSC) and [^99m^Tc]Tc-albumin colloid (TcAC) for SLN detection in the head and neck region.

**Methods:**

Data from 62 patients with cutaneous malignancies of the head who were injected with TcTM, TcSC, or TcAC before SLN imaging (SLN-I) and SLN excision (SLN-E) between 2012 and 2021 were retrospectively analysed. SLN-I was performed using planar lymphoscintigraphy and SPECT/CT, and a gamma probe was used for SLN-E. The SLN-I localisation rate (patients with SLNs) and degree (SLN number) and SLN-E relocalisation rate (patients with SLNs) and ratio (SLN number in SLN-E/SLN number in SLN-I) were compared between TcTM, TcSC, and TcAC.

**Results:**

TcTM showed similar SLN-I localisation rates for primaries in the anterior and posterior head region compared with TcSC (84.6% vs. 72.4%, *p*=0.680; both 100.0%) and TcAC (84.6% vs. 75.0%, *p*=1.000; both 100.0%). The SLN-I localisation degree for TcTM was higher for primaries in the anterior head region and similar for primaries in the posterior head region compared with TcSC (3.2 vs. 2.3, *p*=0.034; and 1.8 vs. 2.2, *p*=0.506) and TcAC (3.2 vs. 2.0, *p*=0.038; and 1.8 vs. 2.7, *p*=0.329). The SLN-E relocalisation rates and ratios were similar for all.

**Conclusion:**

On the basis of a limited study design that compared three different tracers in three different patient groups, TcTM showed comparable overall performance to TcSC and TcAC.

## Introduction

Sentinel lymph node (SLN) biopsy is a widely accepted, minimally invasive procedure used in the management of cutaneous malignancies of the head to stage regional lymph nodes and obtain information for prognosis and further treatment planning [1, 2]. It is associated with a low surgical extent, low postoperative morbidity, and optimised histopathologic examination of small numbers of lymph nodes [3].

As a standard of care, [^99m^Tc]Tc-sulphur colloid (TcSC) and [^99m^Tc]Tc-albumin colloid (TcAC) are used as radiopharmaceuticals for SLN detection [4]. However, both colloids are slowly cleared from the injection site and passively retained in lymph nodes; in part this results in the inability to localise SLNs due to the overlap between SLNs and the injection site or insufficient accumulation of the radiopharmaceutical in SLNs [5–8]. These limitations are particularly evident in the head and neck region due to the close proximity between SLNs and the injection site and the complex lymphatic drainage patterns with multiple small lymph nodes, resulting in SLN localisation rates as low as 65% in some cases [2, 5, 7, 9]. In contrast, [^99m^Tc]Tc-tilmanocept (TcTM) is rapidly cleared from the injection site and actively retained in lymph nodes, as it binds to CD206 receptors on the surface of macrophages and dendritic cells in lymph nodes [6, 10]. The binding of TcTM to dendritic cells in SLNs might be even more beneficial for SLN detection, as dendritic cells are involved in early anti-tumour response in SLNs and differing numbers of dendritic cells in SLNs and non-SLNs could be used for the differentiation between SLNs and non-SLNs [11, 12].

A review addressing SLN detection in breast cancer reported promising results with higher SLN localisation rates (proportion of patients with localised SLN) and degrees (number of SLNs per patient) for TcTM than for TcAC [13]. Data on SLN detection in the head and neck region in the clinical setting of cutaneous malignancies of the head are scarce, and results are inconsistent, with both equal and lower SLN localisation rates and degrees reported for TcTM compared with TcSC [14, 15]. However, these studies did not focus on the head and neck region; did not use single-photon emission computed tomography/computed tomography (SPECT/CT), which has been shown to improve the sensitivity of SLN detection in the head and neck region; and partly used additional dyes for the intraoperative SLN detection [16, 17]. Whether the use of TcTM improves SLN detection in the head and neck region in patients with cutaneous malignancies of the head remains unknown.

The aim of this study was to compare TcTM with TcSC and TcAC for SLN detection in the head and neck region using SPECT/CT in patients with cutaneous malignancies of the head.

## Material and methods

### Study population

This study was approved by the local ethics committee (EK 073 – 22). The study population consisted of 62 patients with cutaneous malignancies of the head who were scheduled for SLN imaging (SLN-I) in the Department of Nuclear Medicine and SLN excision (SLN-E) in the Department of Oral and Maxillofacial Surgery between 2012 and 2021. The inclusion criteria were a histologically diagnosed cutaneous malignancy of the head with clinically and radiologically negative nodal status and planned SLN-I and SLN-E as part of the primary surgical procedure. The exclusion criteria were age less than 18 years; the presence of satellite metastasis, in-transit metastasis, or lymphangiosis carcinomatosa; previous surgical procedures other than excision or incision biopsy in the region of the primary tumour; previous surgical procedures in the neck region; previous irradiation of the head or neck region; the use of dyes in SLN-E; pregnancy; and lactation. Patients whose records were incomplete or missing were also excluded.

Clinical data were obtained from clinical, pathologic, and operative records. The following parameters were recorded: sex, age, body mass index, tumour diagnosis, tumour diameter, tumour invasion depth, tumour location, status of multiple resections before SLN-I, time interval between the resection or probing of the tumour for pathological examination and SLN-I, and time interval between SLN-I and SLN-E.

**Fig. 1:**
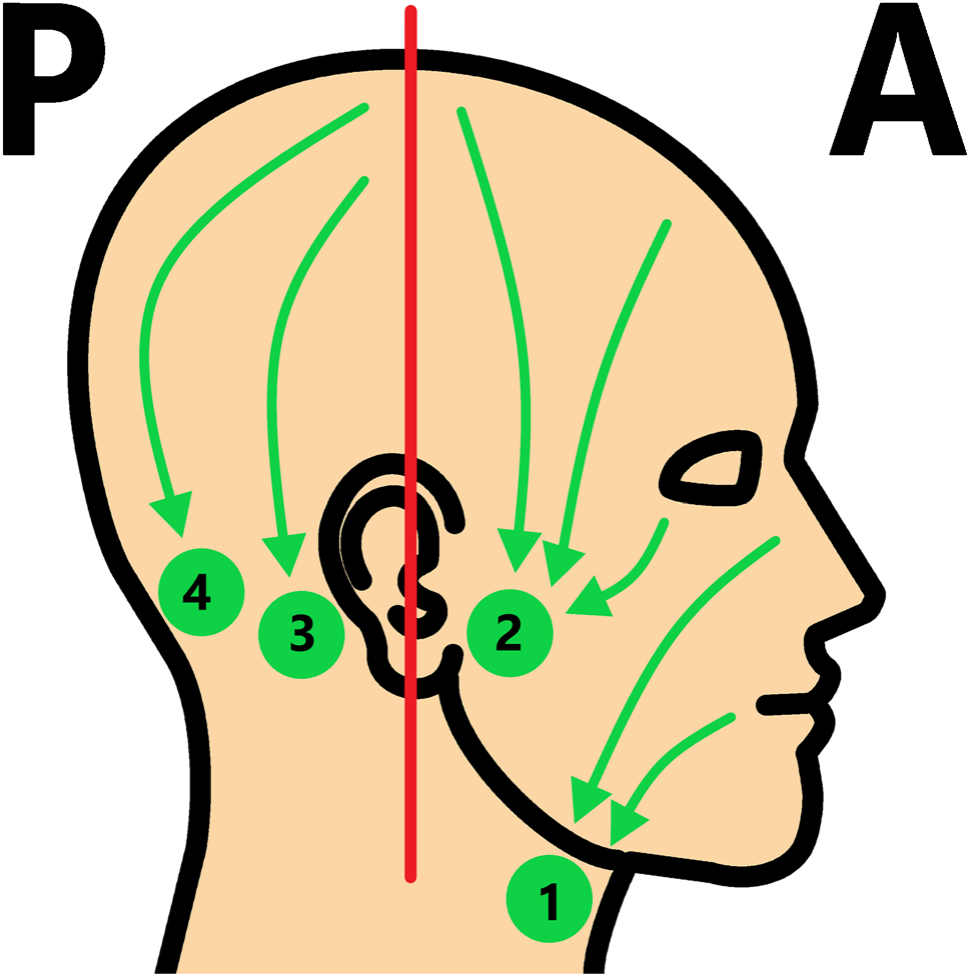
Head regions. Regarding primary tumour location, an anterior (A) and posterior (P) head region was defined based on a vertical landmark line through the external auditory canal, with the anterior region ventral to the landmark line preliminary draining into the submandibular (1) and parotid (2) lymph nodes and the posterior region dorsal to the landmark line preliminary draining into the mastoid (3) and occipital (4) lymph nodes [artwork created with Microsoft Paint, Microsoft, Redmond, USA]

With regard to tumour location, the anterior and posterior head regions were defined based on a vertical landmark line through the external auditory canal. The anterior region was located ventral to the landmark line, with preliminary draining into the submandibular and parotid lymph nodes, and the posterior region was located dorsal to the landmark line, with preliminary draining into the mastoid and occipital lymph nodes (Fig. 1) [18–20].

SLN-I was performed in all patients, and SLN-E was performed in patients with localised SLNs on SLN-I.

### Sentinel lymph node imaging

Planar lymphoscintigraphy (PLS) and SPECT/CT were used for SLN-I in all patients according to institution-specific standard techniques in the Department of Nuclear Medicine. Immediately before the injection, the injection side was cleaned and disinfected with an alcohol swab. All patients received intradermal injections (2–6 doses; each 0.1 mL; mean activity 116.2 MBq) of either TcTM (Lymphoseek, Norgine BV, Amsterdam, The Netherlands), TcSC (Nanocis, CIS Bio, Berlin, Germany), or TcAC (Nanocoll, GE Healthcare, Solingen, Germany; Nanoscan, Radiopharmacy Laboratory, Budaörs, Hungary) around the tumour or scar. Each radiopharmaceutical was prepared according to the manufacturer’s instructions, and all injections were performed within 1 h after radiolabelling.

PLS and SPECT/CT were performed using an Optima NM/CT 640 (GE Healthcare, Chicago, USA) (2018 - 2021; TcTM 10 patients, TcSC 4 patients, TcAC 5 patients) or a Symbia T16 (Siemens Healthineers AG, Erlangen, Germany) (2012 - 2021; TcTM 8 patients, TcSC 30 patients, TcAC 5 patients). When both systems were available, the selection of one system was random and the imaging protocol used was the same throughout the study period.

**Fig. 2:**
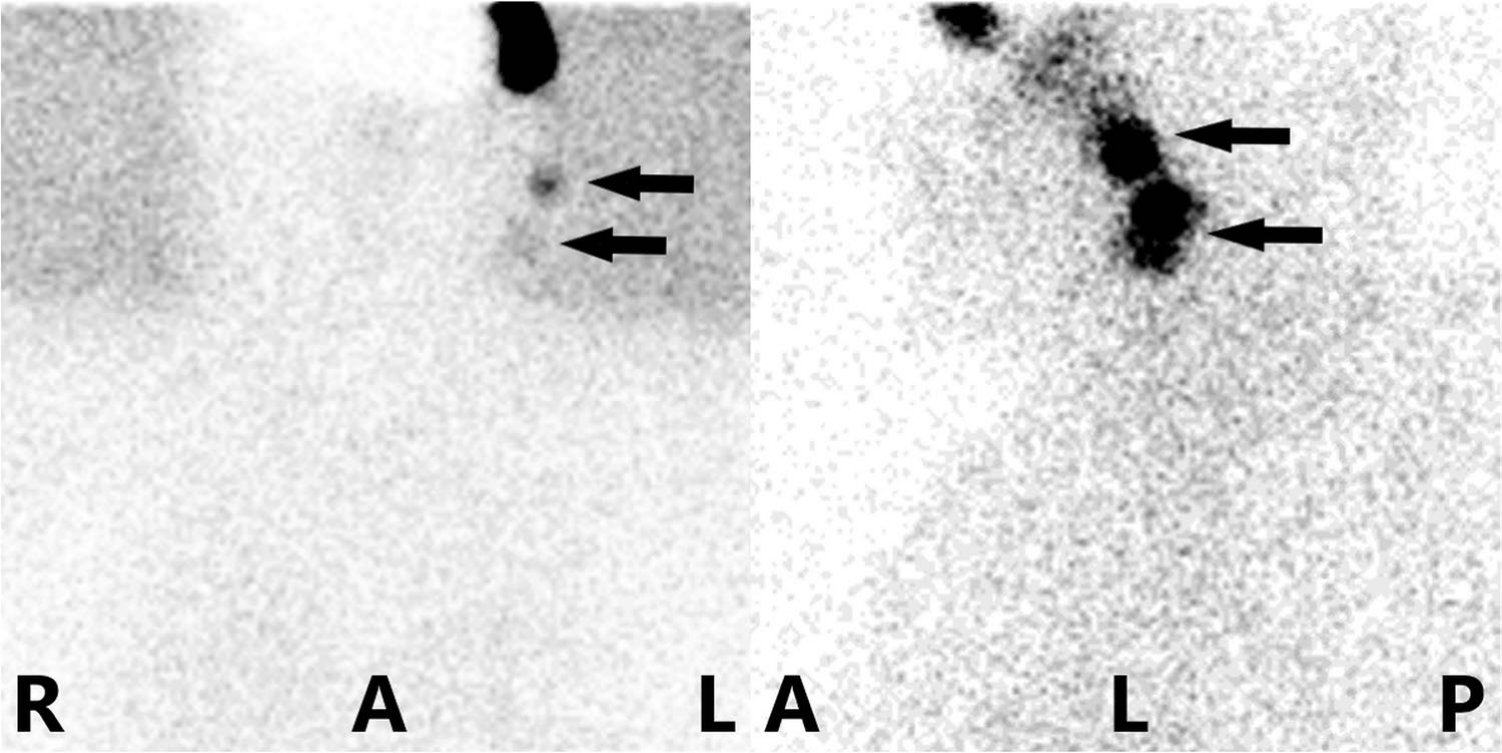
Planar lymphoscintigraphy. Planar lymphoscintigraphy in anterior/posterior projection (left) and lateral projection (right) of a patient with a superior periocular primary tumour 2 h after injection of [^99m^Tc]Tc-tilmanocept; localisation of two SLNs (arrows); abbreviations: R, right patient side; L, left patient side; A, anterior patient side; P, posterior patient side [artwork modified with Microsoft Paint, Microsoft, Redmond, USA]

PLS was performed immediately after injection as early images and subsequently as late images (time interval between 1 and 3 h) in static (1 projection, projection duration 5 min) or dynamic mode (30–60 projections, projection duration 1 min each), in anterior/posterior and lateral projections, with and without lead plates covering the injection sites (Fig. 2). The parameters were as follows: Optima NM/CT 640 256×256 matrix, 2.21-mm pixel size, parallel LEHR collimator; Symbia T16 256×256 matrix, 2.39-mm pixel size, parallel LEHR collimator. In cases in which the SLNs could not be localised in the initial planar images, additional planar images were acquired.

**Fig. 3:**
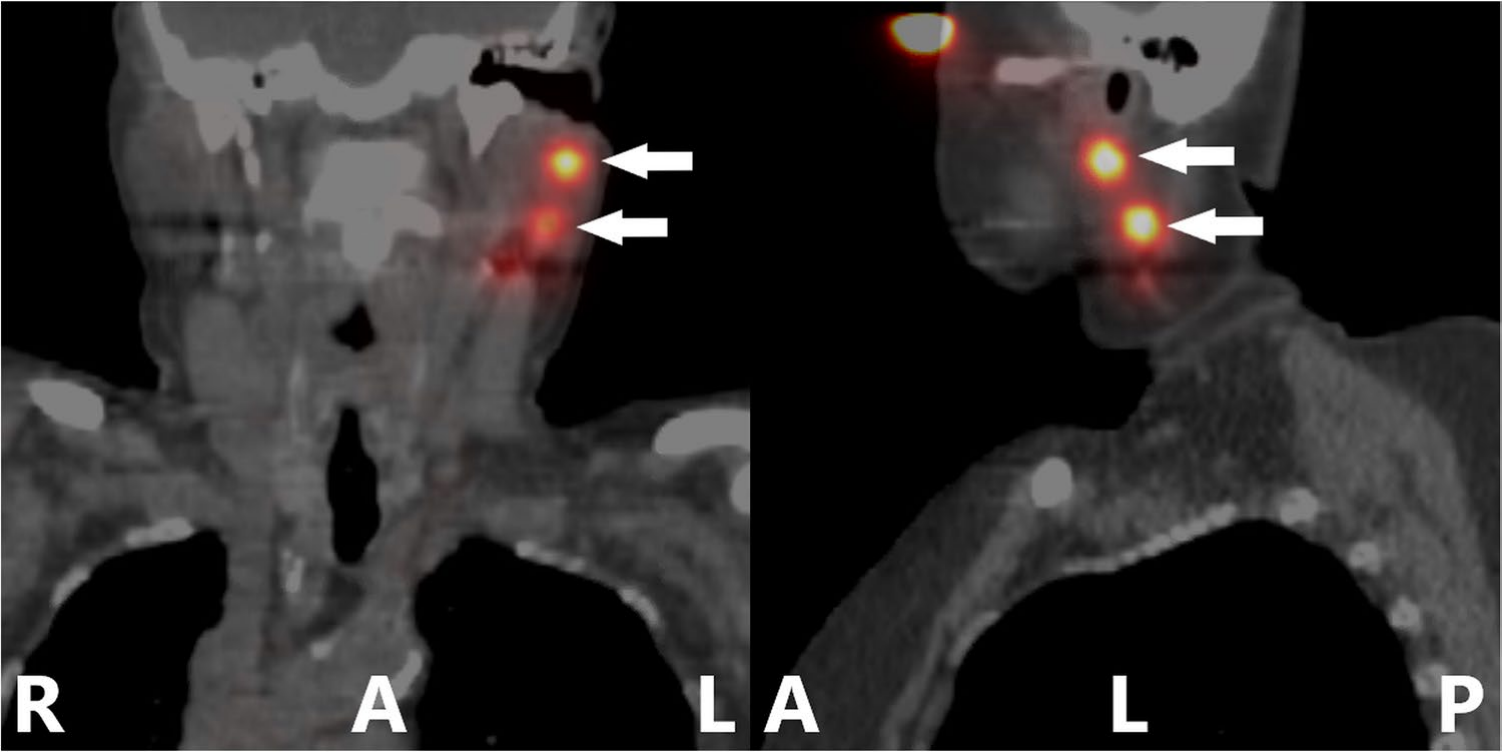
Single-photon emission computed tomography/computed tomography. Single-photon emission computed tomography/computed tomography in coronal projection (left) and sagittal projection (right) of a patient with a superior periocular primary tumour 2 h after injection of [^99m^Tc]Tc-tilmanocept; localisation of two SLNs (arrows); R, right patient side; L, left patient side; A, anterior patient side; P, posterior patient side [artwork modified with Microsoft Paint, Microsoft, Redmond, USA]

SPECT/CT was performed between 1 and 6 h after the PLS (Fig. 3). The parameters for the Optima NM/CT 640 were as follows: SPECT 128×128 matrix, 4.41-mm pixel size, 120 projections, angle 3°, projection duration 24 seconds each, parallel LEHR collimator; CT 4 lines, 2.5-mm slice thickness, 512×512 matrix, 0.97-mm pixel size, 120 kV; Reconstruction 256×256 matrix, 1.95-mm pixel size, 3D OSEM (4 iterations, 10 subsets). The parameters for the Symbia T16 were as follows: SPECT 128×128 matrix, 4.79-mm pixel size, 128 projections, angle 2.8°, projection duration 20 seconds each, parallel LEHR collimator; CT 16 lines, 5.0-mm slice thickness, 512×512 matrix, 0.976-mm pixel size, 130 kV; Reconstruction 128×128 matrix, 4.79-mm pixel size, 3D OSEM (8 iterations, 16 subsets, 9-mm Gaussian filter).

The SLNs were identified and localised by two nuclear physicians, according to the established criteria of the guidelines of the German Society of Nuclear Medicine (i.e.,= the presence of a direct lymphatic vessel tract from the primary site to the lymph node, signal timing of the lymph node, anatomic localisation of the lymph node, and relative uptake of the lymph node) [21].

The radiologic reports were reviewed, and the PLS and SPECT/CT images were examined with respect to the SLN-I localisation rate (i.e. the proportion of patients with localised SLNs on SLN-I in relation to all patients examined with SLN-I) and SLN-I localisation degree (i.e. the number of localised SLNs on SLN-I per patient in patients with localised SLNs on SLN-I). In addition, in relation to the proposed lymphatic drainage pathways described above, the presence of atypically located SLNs was examined.

### Sentinel lymph node excision

SLN-E was performed in all patients who had localised SLNs on SLN-I, using a hand-held gamma probe, according to institution-specific standard techniques in the Department of Oral and Maxillofacial Surgery. In cases without localised SLNs on SLN-I, SLN-E was not performed; either conventional neck dissection or no lymph node excision was performed.

The mean time interval between the radiopharmaceutical injection and SLN-E was 23 h. SLN localisation was based on the definition that a lymph node with a radioactivity count three times higher than that of the background were designated as an SLN. The background measurement was performed by placing the probe tip on the skin surface, at least 20 cm from the injection site. The absence of radioactive lymph nodes defined the failure of SLN localisation. Localised SLNs were extirpated, and after the removal of all SLNs, the surgical area was checked to ensure that no significant residual radioactivity was present. The number of SLNs per patient was recorded according to the designated count by the surgeon in the operation room.

The operation reports were reviewed for the SLN-E relocalisation rate (i.e. the proportion of patients with localised SLN in SLN-E in relation to all patients examined with SLN-E) and SLN-E relocalisation ratio (i.e. the ratio of localised SLNs in SLN-E and localised SLNs on SLN-I per patient in patients with localised SLNs on SLN-I).

### Statistical data analysis

Each patient was categorised into one of three groups according to the radiopharmaceutical used (TcTM, TcAC, or TcSC). Differences in clinical parameters between groups were analysed using the chi-squared test, Fisher’s exact test, or Freeman-Halton test for categorical data and the Mann-Whitney test for metric data. For further analysis, the patients were separated according to the locations of their primary tumours (i.e. those with primary tumours in the anterior head region and those with primary tumours in the posterior head region). Differences between groups in the SLN-I localisation rate and SLN-E relocalisation rate were analysed using the Fisher’s exact test, and differences between groups in the SLN-I localisation degree and SLN-E relocalisation ratio were analysed using the Mann-Whitney test (for the anterior head region, the posterior head region, and both head regions combined). Differences between groups regarding the presence of atypically located SLNs were analysed using the Fisher’s exact test. *P*-values <0.05 were considered statistically significant. The statistical analysis was performed using SPSS version 26 (SPSS, IBM, New York, USA).

## Results

### Study population

The study population consisted of a total of 62 patients (18 patients injected with TcTM, 34 patients injected with TcSC, and 10 patients injected with TcAC) (Table 1). Testing for differences between groups (i.e. TcTM vs. TcSC and TcTM vs. TcAC) showed that the groups did not differ with respect to sex (*p*=0.840 and *p*=0.434, respectively), age (*p*=0.482 and p=0.796, respectively), body mass index (*p*=0.686 and *p*=0.356, respectively), tumour diameter (*p*=0.087 and *p*=0.191, respectively), tumour invasion depth (*p*=0.260 and *p*=0.621, respectively), status of re-resection before SLN-I (*p*=0.723 and *p*=1.000, respectively), time interval between biopsy and SLN-I (*p*=0.939 and *p*=0.689, respectively), or time interval between SLN-I and SLN-E (*p*=0.368 and *p*=0.160, respectively). The patients injected with TcTM and those injected with TcSC differed in terms of tumour diagnosis (*p*=0.007). No difference was observed in terms of tumour diagnosis between patients injected with TcTM and those injected with TcAC (*p*=0.906).

**Table 1:** Clinicopathological characteristics

*Variable*	*TcTM (n=18)*	*TcSC (n=34)*	*p-value*	*TcAC (n=10)*	*p-value*
*Sex (n)*					
Male	9 (50.0%)	18 (52.9%)	0.840	7 (70.0%)	0.434*
Female	9 (50.0%)	16 (47.1%)		3 (30.0%)	
Age (years)	74.0 (23.3)	72.0 (13.5)	0.482	75.0 (23.3)	0.796
BMI (kg/m^2^)	27.1 (5.9)	26.7 (5.2)	0.686	24.7 (8.0)	0.356
Tumour diagnosis (*n*)					
Malignant melanoma	10 (55.6%)	30 (88.2%)	0.007**	7 (70.0%)	0.906**
Merkel cell carcinoma	4 (22.2%)	4 (11.8%)		1 (10.0%)	
Squamous cell carcinoma	3 (16.7%)	0 (0.0%)		2 (20.0%)	
Sebaceous gland carcinoma	1 (5.6%)	0 (0.0%)		0 (0.0%)	
Tumour diameter (mm)	7.0 (9.0)	12.0 (14.3)	0.087	11.0 (12.0)	0.191
Tumour invasion depth (mm)	4.5 (4.7)	2.3 (2.9)	0.260	1.8 (4.7)	0.621
Re-resection before SLN-I (n)					
No	14 (77.8%)	28 (82.4%)	0.723*	8 (80.0%)	1.000*
Yes	4 (22.2%)	6 (17.6%)		2 (20.0%)	
Time interval (biopsy – SLN-I) (days)	37.5 (28.8)	35.5 (22.0)	0.939	38.0 (35.0)	0.689
Time interval (SLN-I – SLN-E) (h)	23.0 (1.3)	22.5 (2.9)	0.368	22.0 (3.3)	0.160

Parameters are indicated as numbers (with percentage) for categorial data (sex, tumour diagnosis, re-resection before SLN-I) or median (with interquartile range) for metric data (age, BMI, tumour diameter, tumour invasion depth, time interval (diagnosis – SLN-I), time interval (SLN-I – SLN-E)) (separately described for the group of patients injected with TcTM, the group of patients injected with TcSC, and the group of patients injected with TcAC); *p*-values corresponding to testing for differences between groups with chi-squared test, Fisher’s exact test (*), or Freeman-Halton test (**) for categorical data or Mann-Whitney test for metric data (TcTM vs. TcSC and TcTM vs. TcAC); significant *p*-values are bold; BMI, body mass index; SLN-I, sentinel lymph node imaging; SLN-E, sentinel lymph node excision; TcTM, [^99m^Tc]Tc-tilmanocept; TcSC, [^99m^Tc]Tc-sulphur colloid; TcAC, [^99m^Tc]Tc-albumin colloid

Primary tumours were located in the anterior head region in 39 patients and in the posterior head region in 23 patients (Table 2).

**Table 2:** Tumour location

*Region*	*TcTM (n=18)*	*TcSC (n=34)*	*TcAC (n=10)*
Anterior head region (*n*)			
Frontal	1 (5.6%)	3 (8.8%)	0 (0.0%)
Parietotemporal anterior	4 (22.2%)	4 (11.8%)	1 (10.0%)
Auricular anterior	0 (0.0%)	0 (0.0%)	0 (0.0%)
Preauricular	0 (0.0%)	2 (5.9%)	0 (0.0%)
Periocular	2 (11.1%)	1 (2.9%)	0 (0.0%)
Infraorbital	3 (16.7%)	3 (8.8%)	0 (0.0%)
Nasal	0 (0.0%)	1 (2.9%)	1 (10.0%)
Perioral	3 (16.7%)	0 (0.0%)	1 (10.0%)
Buccal	0 (0.0%)	8 (23.6)	1 (10.0%)
Posterior head region (*n*)			
Occipital	1 (5.6%)	2 (5.9%)	2 (20.0%)
Parietotemporal posterior	1 (5.6%)	4 (11.8%)	2 (20.0%)
Auricular posterior	1 (5.6%)	6 (17.6%)	2 (20.0%)
Retroauricular	2 (11.1%)	0 (0.0%)	0 (0.0%)

Parameters are indicated as numbers (with percentage) (separately described for the group of patients injected with TcTM, the group of patients injected with TcSC, and the group of patients injected with TcAC); TcTM, [^99m^Tc]Tc-tilmanocept; TcSC, [^99m^Tc]Tc-sulphur colloid; TcAC, [^99m^Tc]Tc-albumin colloid

### Localisation rate and degree for sentinel lymph node imaging

**Fig. 4:**
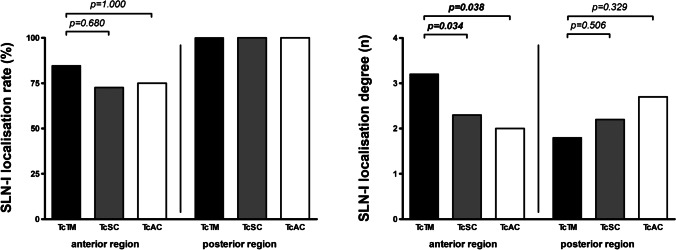
SLN-I localisation rate and degree. Each column represents the SLN-I localisation rate (percent) (left) or mean SLN-I localisation degree (number) (right) for TcTM, TcSC, and TcAC (separately described for the anterior and posterior head region); *p*-values corresponding to testing for differences with Fisher’s exact test (SLN-I localisation rate) or Mann-Whitney test (SLN-I localisation degree); significant *p*-values are bold; SLN-I, sentinel lymph node imaging; TcTM, [^99m^Tc]Tc-tilmanocept; TcSC, [^99m^Tc]Tc-sulphur colloid; TcAC, [^99m^Tc]Tc-albumin colloid [artwork created with GraphPad Prism 4, GraphPad Software, San Diego, USA]

No difference in the SLN-I localisation rate was observed between patients injected with TcTM and patients injected with TcSC for primary tumours in both head regions combined (88.9% vs. 82.4%, *p*=0.698) or for primary tumours in the anterior and posterior head regions separately (84.6% vs. 72.2%, *p*=0.680; and 100.0% vs. 100.0%, *p*-value not available; respectively) (Table 3, Fig. 4). Furthermore, no difference in the SLN-I localisation rate was observed between patients injected with TcTM and patients injected with TcAC for primary tumours in both head regions combined (88.9% vs. 90.0%, *p*=1.000) or for primary tumours in the anterior and posterior head regions separately (84.6% vs. 75.0%, *p*=1.000; and 100.0% vs. 100.0%, *p*-value not available; respectively). For primary tumours in the anterior head region, patients injected with TcTM had a higher SLN-I localisation degree than patients injected with TcSC or TcAC (3.2 vs. 2.3, *p*=0.034; and 3.2 vs. 2.0, *p*=0.038; respectively). These differences between patients were not observed for primary tumours in both head regions combined or separately for primary tumours in the posterior head region. Atypically located SLNs were found in a total of 12 out of 62 patients (19.4%). No difference in the presence of atypically located SLNs was observed between patients injected with TcTM and TcSC (27.8% vs. 14.7%; *p*=0.287) and TcTM and TcAC (27.8% vs. 20.0%; *p*=1.000).

**Table 3:** SLN-I localisation and SLN-E relocalisation

*Variable*	*TcTM (n=18)*	*TcSC (n=34)*	*p-value*	*TcAC (n=10)*	*p-value*
*Anterior + posterior head regions (n=62)*					
SLN-I localisation rate (%)	88.9%	82.4%	0.698	90.0%	1.000
SLN-I localisation degree (*n*; mean (SD))	2.8 (1.1)	2.2 (1.2)	0.093	2.4 (1.2)	0.357
SLN-E relocalisation rate (%)	87.5%	78.6%	0.689	88.9%	1.000
SLN-E relocalisation ratio (%; mean (SD))	85.4% (34.6%)	76.2% (42.2%)	0.581	88.9% (33.3%)	0.803
*Anterior head region (n=39)*					
SLN-I localisation rate (%)	84.6%	72.7%	0.680	75.0%	1.000
SLN-I localisation degree (*n*; mean (SD))	3.2 (0.9)	2.3 (1.4)	**0.034**	2.0 (0.0)	**0.038**
SLN-E relocalisation rate (%)	81.8%	81.3%	1.000	66.7%	1.000
SLN-E relocalisation ratio (%; mean (SD))	78.8% (40.2%)	77.1% (41.7%)	1.000	66.7% (57.7%)	0.885
*Posterior head region (n=23)*					
SLN-I localisation rate (%)	100.0%	100.0%	-	100.0%	-
SLN-I localisation degree (*n*; mean (SD))	1.8 (1.1)	2.2 (0.9)	0.506	2.7 (1.5)	0.329
SLN-E relocalisation rate (%)	100.0%	75.0%	0.515	100.0%	-
SLN-E relocalisation ratio (%; mean (SD))	100.0% (0.0%)	75.0% (45.2%)	0.442	100.0% (0.0%)	1.000

Parameters are indicated as absolute percentage (SLN-I localisation rate and SLN-E relocalisation rate) or mean number or percentage (with standard deviation) (SLN-I localisation degree and SLN-E relocalisation ratio) (separately described for the group of patients injected with TcTM, the group of patients injected with TcSC, and the group of patients injected with TcAC); calculation of SLN-I localisation degree, SLN-E relocalisation rate, and SLN-E relocalisation ratio included only patients with successful SLN-I localisation; p-values corresponding to testing for differences between groups with Fisher’s exact test (SLN-I localisation rate and SLN-E relocalisation rate) and Mann-Whitney test (SLN-I localisation degree and SLN-E relocalisation ratio) (TcTM vs. TcSC and TcTM vs. TcAC); significant p-values are bold; SLN-I, sentinel lymph node imaging; SLN-E, sentinel lymph node excision; TcTM, [^99m^Tc]Tc-tilmanocept; TcSC, [^99m^Tc]Tc-sulphur colloid; TcAC, [^99m^Tc]Tc-albumin colloid

### Relocalisation rate and ratio for sentinel lymph node excision

**Fig. 5:**
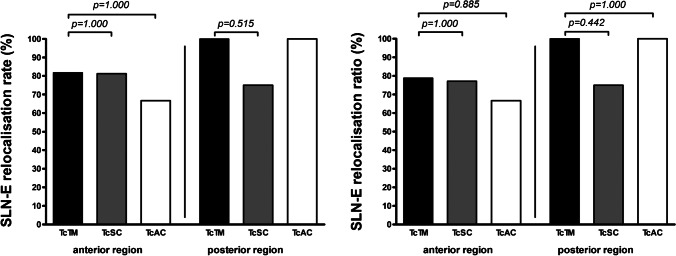
SLN-E relocalisation rate and ratio. Each column represents the SLN-E relocalisation rate (percent) (left) or mean SLN-E relocalisation ratio (percent) (right) for TcTM, TcSC, and TcAC (separately described for the anterior and posterior head region); *p*-values corresponding to testing for differences with Fisher’s exact test (SLN-E relocalisation rate) or Mann-Whitney test (SLN-E relocalisation ratio); significant *p*-values are bold; SLN-E, sentinel lymph node excision; TcTM, [^99m^Tc]Tc-tilmanocept; TcSC, [^99m^Tc]Tc-sulphur colloid; TcAC, [^99m^Tc]Tc-albumin colloid [artwork created with GraphPad Prism 4, GraphPad Software, San Diego, USA]

No difference in the SLN-E relocalisation rate was observed between patients injected with TcTM and patients injected with TcSC for primary tumours in both head regions combined (87.5% vs. 78.6%, *p*=0.689) or for primary tumours in the anterior and posterior head regions separately (81.8% vs. 81.3%, *p*=1.000; and 100.0% vs. 75.0%, *p*=0.515; respectively) (Table 3, Fig. 5). Furthermore, no difference in the SLN-E relocalisation rate was observed between patients injected with TcTM and patients injected with TcAC for primary tumours in both head regions combined (87.5% vs. 88.9%, *p*=1.000) or for primary tumours in the anterior and posterior head regions separately (81.8% vs. 66.7%, p=1.000; and 100.0% vs. 100.0%, *p*-value not available; respectively). No differences were observed between patients injected with TcTM or TcSC or between patients injected with TcTM or TcAC in terms of the SLN-E relocalisation ratio for primary tumours in both head regions combined or for primary tumours in the anterior and posterior head regions separately. No additional SLNs were found at SLN-E that were not mapped on SLN-I.

## Discussion

As an alternative to elective neck dissection, SLN biopsy is an emerging, minimally invasive procedure for the staging of regional cervical lymph nodes in patients with clinically and radiologically negative nodal status undergoing surgical treatment for cutaneous malignancies of the head [2, 8, 22–25]. It relies on both the orderly spread of tumour cells from the primary tumours to the SLNs and the low likelihood of subsequent metastatic nodes in cases of non-metastatic SLNs [18, 22–24]. However, complex lymphatic drainage patterns, a high likelihood of overlap between SLNs and the injection sites due their close proximity, and insufficient accumulation of the radiopharmaceutical due to multiple small lymph nodes predispose the head and neck region to SLN biopsy failure [2, 5, 7–9, 26]. In view of the adverse consequences of SLN failure for patients, such as undertreatment due to the unawareness of metastatic lymph nodes or overtreatment with unnecessary neck dissection, improving the detection of SLNs in the head and neck region is of high clinical importance [18].

In this study, the performance of TcTM for the detection of SLNs in the head and neck region in patients with cutaneous malignancies of the head was evaluated using PLS and SPECT/CT and compared to the performance of TcSC and TcAC in terms of key parameters, such as the SLN localisation rate and localisation degree [27, 28]. The study protocol used was similar to the recently described study protocol presented in the guidelines for SLN localisation in oral cavity squamous cell carcinoma [29]. To account for the different particle sizes, TcTM was compared with TcSC and TcAC separately [25]. To account for the regionally varying difficulties in SLN detection in the head and neck region (e.g. the overlap between SLNs and the injection sites due their close proximity, which is particularly encountered in the localisation of submandibular SLNs, and the insufficient accumulation of radiopharmaceuticals in SLNs due to multiple small SLNs, which is particularly encountered in the localisation of parotid SLNs), the head region was divided into an anterior and posterior region in terms of the primary tumour location [5, 6, 9]. Indeed, the SLN-I localisation rate differed between the anterior and posterior head regions (76.9% vs. 100.0%, Fisher’s exact test *p*=0.020).

This study showed similar SLN-I localisation rates for TcTM, TcSC, and TcAC for primary tumours in both head regions combined and for primary tumours in the anterior and posterior head regions separately. The results are consistent with those from two previous studies that partially included some patients with cutaneous malignancies of the head [15, 30]. Those studies reported similar SLN-I localisation rates for TcTM and TcSC (98% and 99%, respectively, in one study, and 97% and 98%, respectively, in the other study).

However, the SLN-I localisation rates for TcTM and TcSC were lower in our study. This may be due to the fact that our study included only patients with cutaneous malignancies of the head, and SLN localisation in the head and neck region is difficult [2, 5–9]. Interestingly, the SLN-I localisation rates were lower for all radiopharmaceuticals for primary tumours in the anterior head region only. This may be explained by the even more pronounced difficulties in SLN localisation in the anterior head region [2, 5, 6, 8, 9]. Given that the properties of TcTM (e.g. rapid injection site clearance and active binding properties in SLNs) are presumably more suitable for SLN localisation, a higher SLN localisation rate would have been expected for TcTM than for TcSC or TcAC; furthermore, an even greater difference between TcTM and TcSC would have been expected due to the greater particle size of TcSC, which impedes injection site clearance [6, 10, 25]. However, the groups of patients injected with TcTM or TcSC differed with respect for tumour diagnosis, thus the lack of difference between the radiopharmaceuticals may have been due to the influences of differences in cancer biology on peripheral lymph vessels [31]. In contrast, an influence of the status of re-resection prior to SLN-I and the time interval between biopsy and SLN-I by impairment of the functionality of lymph vessels around the injection site — for example, by tissue fibrosis — seems unlikely, as the groups of patients injected with TcTM, TcSC, or TcAC did not differ with respect to these parameters.

This study showed a higher SLN-I localisation degree for primary tumours in the anterior head region, and similar SLN-I localisation degrees for primary tumours in both head regions combined and for primary tumours in the posterior head region, for TcTM compared to TcSC and TcAC. Two previous studies, which partially included patients with cutaneous malignancies of the head, showed equal SLN-I localisation degrees for TcTM and TcSC [15, 30]. The higher SLN-I localisation degree for TcTM than for TcSC and TcAC for primary tumours only in the anterior head region may indicate that the presumably more suitable properties of TcTM for SLN localisation, such as its rapid injection site clearance and active binding properties in SLNs, become even more effective in the anterior head region [6, 10]. However, differences in anatomic SLN numbers and primary localisation between patients, in addition to individual differences in radiopharmaceutical uptake rates, may also contribute to differing SLN-I localisation degrees [32–35].

The similar SLN-E relocalisation rates and ratios for TcTM, TcSC, and TcAC suggest that radiopharmaceutical accumulation sufficient for SLN-I localisation persists until SLN-E for all of these radiopharmaceuticals; this is consistent with the same SLN-E localisation rates for TcTM, TcSC, and TcAC that have been observed in breast cancer studies [36–38]. This implies that the retention of TcSC and TcAC in SLNs by phagocytosis and thus independent of binding properties is also sufficient for SLN-I and SLN-E [2].

In this study, atypically located SLNs were found in approximately 20% of all cases, which is consistent with other studies addressing SLN detection in the head and neck region and may be due to the great variability of lymphatic drainage in the head and neck region [18, 19, 33, 34]. However, no differences were found between TcTM, TcSC, and TcAC in the presence of atypically located SLNs.

In addition to SLN detection, the binding of TcTM to dendritic cells in SLNs may have further implications for tumour diagnosis and therapy. Considering the key role of dendritic cells in initiating an anti-tumour response after initial contact with tumour antigen in SLNs, altered dendritic cell numbers in SLNs and consequently altered TcTM accumulation in SLNs could reflect tumour activity and immune response [12, 39].

The limitations of this study include its retrospective design and the heterogeneity of the study population, for example, in terms of the time interval between radiopharmaceutical injection and imaging and the small number of patients. However, SLN localisation rates and degrees should not have been affected by such heterogeneity, because additional images were obtained in cases in which SLN localisation failed on early imaging; additionally, the exclusive focus of this study on cutaneous malignancies of the head may explain the small number of patients. Furthermore, differences in experience between nuclear physicians in SLN-I and surgeons in SLN-E cannot be excluded; however, valid procedural standards likely mitigated the effects of such differences. In addition, the two different imaging systems used during the study period were not applied equally in all patient groups, so that an influence of the imaging system on the results cannot be excluded. In general, it should be mentioned that comparing three different radiopharmaceuticals by comparing three groups of patients, each injected with one radiopharmaceutical, limits the generalizability and the level of evidence obtained from this study. However, a study design in which all three radiopharmaceuticals are used consecutively in the same patient is not suitable due to the non-repeatability of the surgical procedure.

This study was the first to evaluate the performance of TcTM in SLN detection using PLS and SPECT/CT in the head and neck region for cutaneous malignancies of the head and to compare the performance of TcTM with those of the commonly used TcSC and TcAC. The SLN-I localisation rates, SLN-E relocalisation rates, and SLN-E relocalisation ratios were similar for TcTM, TcSC, and TcAC. This precludes the general use of TcTM instead of TcSC or TcAC for SLN detection in the head and neck region in patients with cutaneous malignancies of the head due to the higher cost of TcTM [15, 40]. However, the SLN-I localisation degree was higher with TcTM than with TcSC or TcAC for primary tumours in the anterior head region. Studies have shown that a higher SLN-I localisation degree, and, consequently, a higher number of SLNs identified and removed, is associated with a lower false-negative rate of SLN biopsy in the head and neck region for cutaneous malignancies of the head [41, 42]. In addition, a higher SLN-I localisation degree of TcTM may be considered beneficial in terms of an only minimally increased extent of surgical intervention and postoperative morbidity (due to the higher number of SLNs removed) relative to the tremendous negative impact on outcomes in cases of overlooked metastatic SLNs [15, 23, 27, 28, 30].

Therefore, based on the results of this retrospective, exploratory study, the use of TcTM should only be recommended for SLN detection in the head and neck region in patients with cutaneous malignancies in the anterior head region [15, 40].

Further studies are needed to confirm whether a higher SLN-I localisation degree is associated with only a moderate increase in postoperative morbidity (therefore preserving the benefits of SLN biopsy), whether a higher SLN-I localisation degree is associated with a lower false-negative rate of SLN biopsy in patients with cutaneous malignancies in the anterior head region, and whether our results can be confirmed in a larger study population.

## Data Availability

The datasets generated and analysed during the current study are available from the corresponding author on reasonable request.
